# Prognostic Evaluation of Vimentin Expression in Correlation with Ki67 and CD44 in Surgically Resected Pancreatic Ductal Adenocarcinoma

**DOI:** 10.1155/2017/9207616

**Published:** 2017-03-22

**Authors:** Despoina Myoteri, Dionysios Dellaportas, Panagis M. Lykoudis, Alexandros Apostolopoulos, Athanasios Marinis, Adamantia Zizi-Sermpetzoglou

**Affiliations:** ^1^Pathology Department, “Tzaneion” General Hospital, Pireaus, Greece; ^2^2nd Department of Surgery, University Hospital “Aretaeion”, Athens, Greece; ^3^Division of Surgery & Interventional Science, University College London, London, UK; ^4^1st Department of Surgery, “Tzaneion” General Hospital, Pireaus, Greece

## Abstract

*Purpose.* Radical surgical resection with adjuvant chemotherapy or chemo-radiotherapy is the most effective treatment for pancreatic ductal adenocarcinoma (PDAC). However, relatively few studies investigate the prognostic significance of biological markers in PDAC. This study aims to look into the expressions of vimentin, Ki67, and CD44 in PDAC surgical specimens and their potential prognostic implications in survival. *Method.* The study was designed as retrospective, and vimentin, Ki67, and CD44 expressions were evaluated by immunohistochemistry in 53 pancreatic ductal adenocarcinoma cases. Overall survival was assessed by the Kaplan–Meier method. *Results.* Patients' median age was 68 years. The median survival was 18 months. The tumors were T3-4 in 40/53 (75.5%), and metastases in lymph nodes were found in 42 out of 53 (79.2%) cases. On multivariate analysis, the size of primary tumor (*p* < 0.001), the surgical resection margin status (*p* = 0.042), and vimentin expression (*p* = 0.011) were independently correlated with overall survival. *Conclusions.* Long-term survival after resection of PDAC is still about 15%. Vimentin expression is a potential independent adverse prognostic molecular marker and should be included in histopathological reports. Also, CD44 expression correlates with high Ki67, vimentin positivity, and N stage and may represent a potential target of novel therapeutic modalities in pancreatic adenocarcinoma patients.

## 1. Introduction

Pancreatic ductal adenocarcinoma (PDAC) is one of the most aggressive malignancies, with an estimated overall 5-year survival rate of less than 5% in all stages [1]. The cornerstone of treatment is surgical resection of the tumor in patients with disease amenable to surgery. However, the latter group represents the minority of cases, estimated as 10–15% of all PDAC patients. The vast majority of patients usually present with either locally unresectable disease or with distant metastases at the time of diagnosis [2].

Several studies have highlighted potentially histopathological prognostic factors of PDAC [3]. Established adverse prognostic factors are tumor size (>3 cm), high tumor grade, lymph nodal metastases, and microvascular and perineural invasion [4].

Molecular analysis is another promising source of clinically useful prognostic biomarkers. These could be involved in pancreatic tumor growth, apoptosis, angiogenesis, invasion, and resistance to chemotherapy [5]. For instance, p27, p53, transforming growth factor *β*1, Cox-2, CD34, S100A4, CD44, and human equilibrative nucleoside transporter are already suggested as such biomarkers [6, 7].

The purpose of the present study was to investigate vimentin expression in PDAC and to explore its association with clinicopathological features, CD44 immunoreactivity, Ki67 expression, and long-term clinical outcomes.

## 2. Materials and Methods

This was a retrospective study, of fifty-three consecutive patients who underwent primary surgical resection for PDAC in a single institution over a nine-year period, between the years 2007 and 2015. All data were retrieved from hospital's inpatient records, outpatient's clinic visits documentation, and histopathological and follow-up radiological imaging reports.

All patients had histologically confirmed PDAC. None of the patients had received neoadjuvant chemotherapy or radiotherapy. The analyzed clinicopathological parameters were age (years), sex, tumor size (cm) on final histopathological examination, tumor grade, lymph nodal status, lymphovascular invasion, perineural or intraneural invasion, and final histopathological tumor stage (TNM/AJCC 7th Edition). Overall survival was calculated from the date of surgery to the last follow-up or to patient's death. Median follow-up was 16.2 months, while three patients with early perioperative death (within 30 days of surgery) were not included in the statistical analysis. Postoperative surgical and medical complications were the cause of these.

Slides were obtained from paraffin-embedded tissue block of primary tumor specimen. The sections were reviewed by two consultant histopathologists. For every case, one paraffin block with tumor was selected for the immunohistochemical detection of vimentin, Ki67, and CD44 expressions.

The immunoperoxidase method was performed in three steps using an EnVision Dako kit (Glostrup, Denmark). Omission of primary antibody served as negative control. The antibodies which were used in this study are presented in Table 1.

Immunoreactivity for vimentin was evaluated according to the percentage of tumor cells with positive cytoplasmic staining. Intensity of reaction to vimentin antibody in tumor epithelial cells ranged from weak to strong positive. In most cases, positive tumor cells were scattered diffusely throughout the section examined, constituting at least 10% and in most cases 50% or more of the tumor cells. Tumors were considered to be positive when characterized at least 10% of the neoplastic cells. A cut-off < 10% of positive tumor cells was used to define negative cases [8–10]. The expression of vimentin in stromal fibroblasts was considered as internal control. The immunoreactive stroma serves as a built-in positive control.

In order to evaluate CD44 immunostaining, tumor cells showing membranous staining were regarded as positive. A cut-off < 10% of positive neoplastic cells was used to define low expression, 11–50% to define moderate expression, and >50% to define high expression [11]. In the normal pancreatic parenchyma, the cell surface CD44 immunoreactivity was negative. CD44 expression was found in some lymphocytes and macrophages and was used as internal control [12].

Immunoreactivity for Ki67 was evaluated semiquantitatively and according to the percentage of positive tumor nuclei and was classified as follows: negative, low Ki67 for tumors showing <15% of immunostained nuclei and “high Ki67” for tumors with nuclear immunoreactivity in >16% of tumor cells [10].

Unifactorial analysis between scale and binomial variables was performed using the Mann-Whitney *U* test scale. Multinomial variables were compared using Independent Samples Median test. Correlations of categorical variables in 4-fold tables was performed using Fisher's exact test (2-tailed) and in >4-fold table using chi-square test (2-tailed). Comparisons of scale variables (excluding overall survival) were made using Spearman's correlation test (2-tailed). Overall survival was assessed across groups of categorical variables using the Kaplan–Meier method with both log rank and Breslow tests of significance. Multifactorial analysis of overall survival included independent variables that demonstrated at least statistically suggestive correlation (*p* < 0.1) in unifactorial analysis and was performed using Cox regression, following the “forward stepwise (LR)” method. A *p* value of less than 0.05 was considered statistically significant. Statistical processing of data was conducted using SPSS v20 software (IBM Corporation, Chicago, IL, USA).

## 3. Results

**Figure 1:**
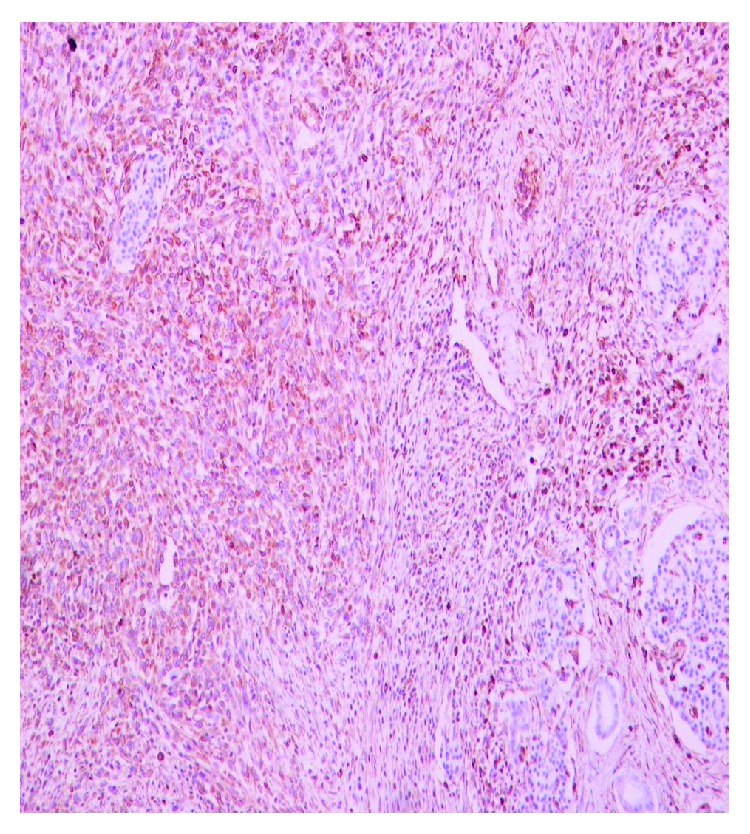


**Figure 2:**
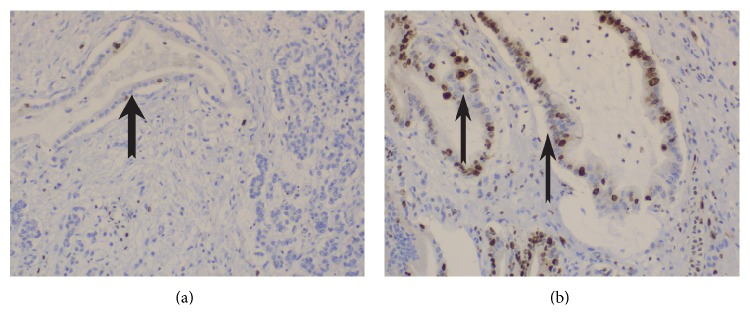


**Figure 3:**
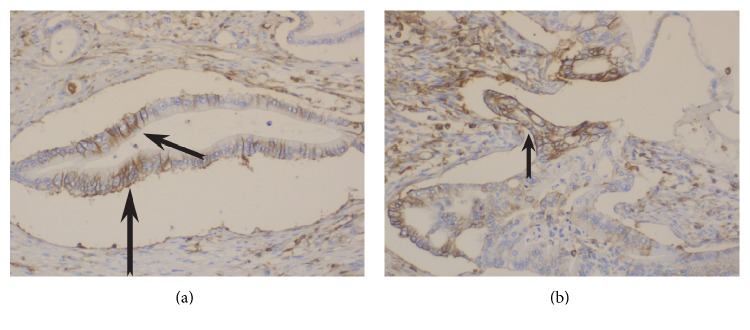


The demographic and histopathological characteristics of included patients are presented in Table 2. Median overall survival is 18 months (range: 6–98 months) while 7 patients (13.2%) are alive on the last follow-up during collection of data. The expression rates of the studied markers are presented in Table 3. Characteristics of samples of immunostaining for vimentin, CD44, and Ki67 are shown in Figures 1, 2 and 3.

**Figure 4:**
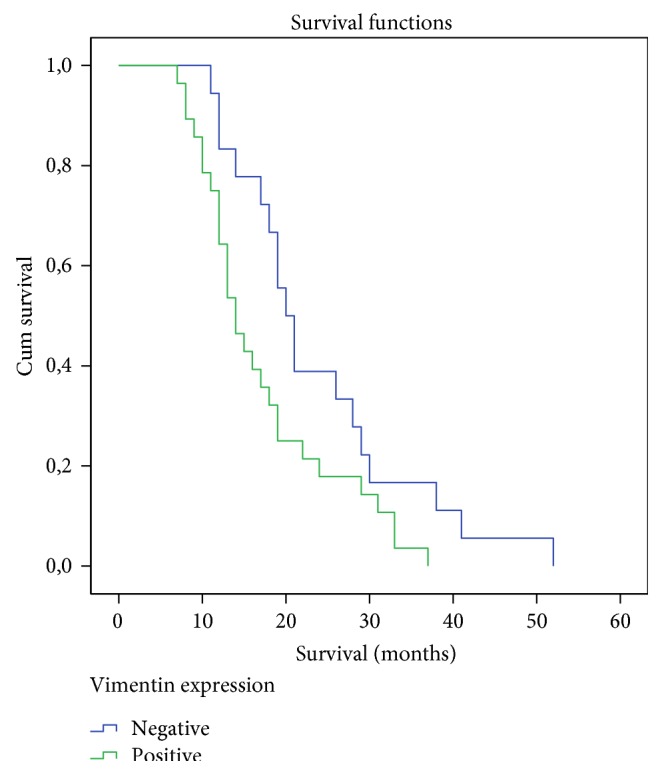


The statistical analysis showed that expression of vimentin is associated with poor histological differentiation independently of the percentage of positive neoplastic cells. Additionally, there is no statistical difference between vimentin expression and primary tumor size. By univariate survival analysis, patient's overall survival was closely related to size, differentiation, and status of lymph nodes as well as microscopic or macroscopic surgical resection margins involvement (R1 or R2 resections), all of which are clinically established prognostic parameters. Multivariate analysis in the group of patients having >10% of neoplastic cell expression, meaning positive for vimentin, showed that this group of patients had a shorter postsurgical survival rate (*p* = 0.035) independent of any other confounding factor, making the expression of vimentin an independent prognostic factor in this study (Figure 4).

Regarding CD44, low expression (<10% of neoplastic cells) was present in 12 cases (22.6%), moderate expression (11–50% of neoplastic cells) in 8 cases (15.1%), and high expression (>50% of neoplastic cells) in 33 cases (62.3%). Only moderate and high expressions were considered as positive.

**Figure 5:**
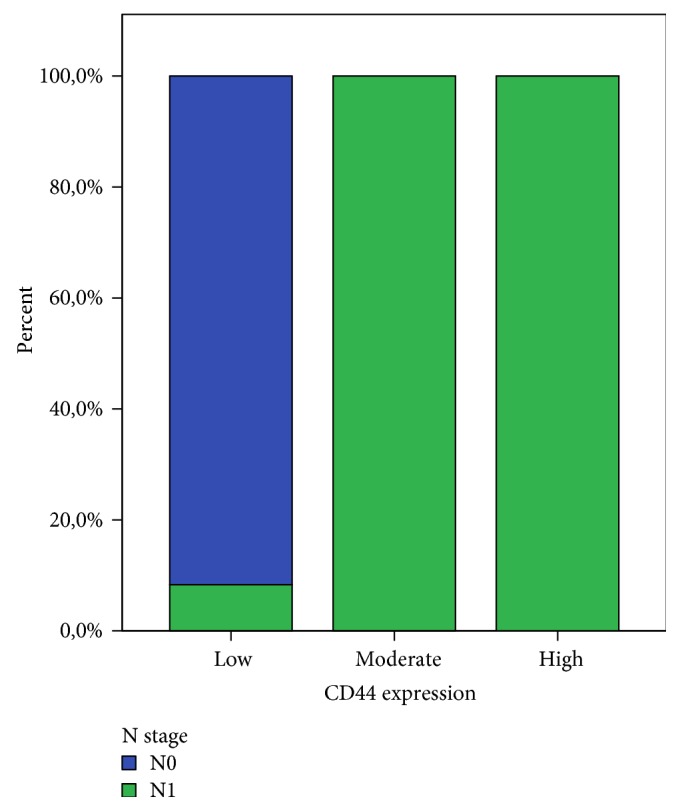


The relationship between CD44 expression and the final histopathological lymph nodal (N) status is shown in Figure 5. On the latter, it is clearly demonstrated that CD44 positivity is significantly correlated with positive lymph nodes on final histopathology.

Ki67 expression was detected in nearly all the tumor sections (52/53). Ki67 does not show reaction to normal pancreatic duct and is therefore used as internal negative control.

**Figure 6:**
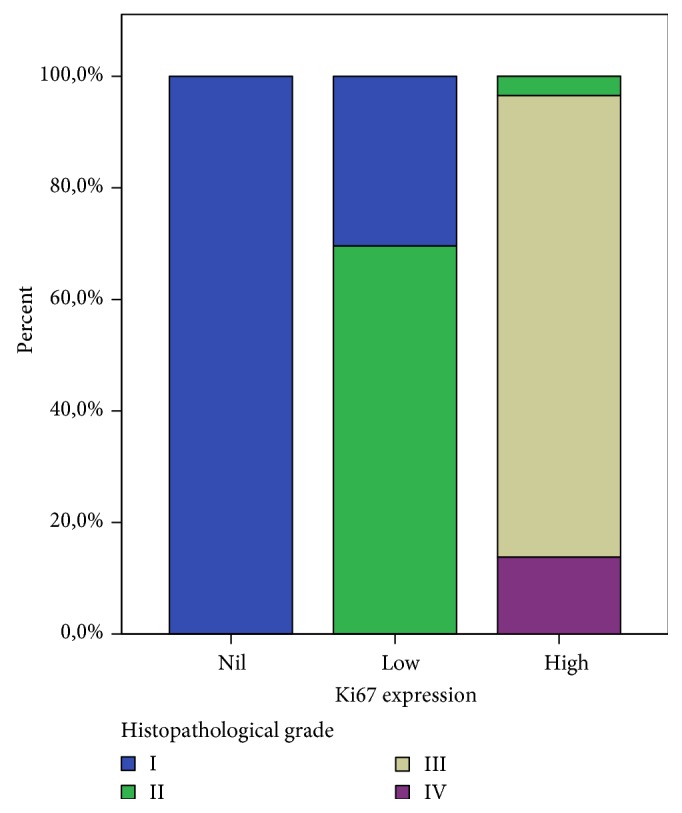


High Ki67 expression (29/53, 54.7%) correlates with tumor grade (Figure 6).

Neither CD44 nor Ki67 demonstrated a statistically significant correlation with survival, thus vimentin remained the only predictive factor (Table 4). The correlation of biomarkers with each other is shown in Table 5.

## 4. Discussion

PDAC is the fourth leading cause of cancer-related deaths worldwide in both men and women [1, 8]. Prognosis remains dismal, as 5-year survival rate is approximately 5% for all stages, reaching 20% for loco-regional tumors and being less than 1% for patients with metastatic disease [13]. Radical surgical resection is the mainstay for curative treatment, although only in about 15% of patients the operative approach is feasible. No or minimal response to chemotherapy or radiotherapy is the norm. All the above indicate that there is a high demand to explore molecular biomarkers and targets which may help to expedite detection, guide treatment, and sequentially improve survival of pancreatic cancer patients [14].

Vimentin is regarded as the main intermediate filament protein of normal mesenchymal tissue [15]. Its role is to sustain cellular integrity and grant resistance against stress factors [16]. Vimentin expression has been detected in epithelial malignancies like prostate, breast, and lung cancer as well as in gastrointestinal and central nervous system tumors. It is well known that epithelial neoplasms with markers of mesenchymal differentiation can induce unusual biological and clinical behavior [17]. More specifically, the overexpression of vimentin in cancer cells is an adverse prognostic factor, correlating with accelerated tumor growth. It is also stated that vimentin can be used to determine the degree of cell differentiation [18]. Vimentin expression in precursor pancreas duct-like cells is detected early in rodent embryos and reaches peak levels soon after birth. This event is most likely due to upregulation of TGF-*β* protein during embryonic period.

Recent studies are claiming that vimentin is a marker of “epithelial/mesenchymal” transdifferentiating (EMT) [19]. The latter seems to be a well-described feature in a variety of tumors [20]. From the histopathological point of view, during EMT, epithelial cells lose their characteristic cell-cell adhesion structures. They also change their polarity; modulate the organization of their cytoskeletal systems; switch expression from keratin- to vimentin-type intermediate filaments; and become isolated, motile, and resistant to anoikis [19].

It has to be mentioned that the differential diagnosis between chronic pancreatitis and pancreatic cancer cannot be based on expression of vimentin. This is because vimentin can be expressed not only in pancreatic cancer cell but also in stromal fibroblasts [19].

In our study, vimentin expression was observed in twenty-eight out of fifty-three (52.8%) cases. A correlation between vimentin expression and poor histological differentiation was revealed. Similar results can be found in other large studies. Moreover, there was no correlation between vimentin expression in neoplastic cells and T stage. The main outcome though of this study is the independent correlation on multivariate analysis of vimentin expression and worse overall survival. This finding is in accordance with previous studies [21]. The diminished overall survival on this latter study [21], for PDAC patients with positive vimentin expression, seems to be an independent of other variable parameters. This is also underlying mesenchymal and poor differentiation.

CD44 is a transmembrane glycoprotein, encoded on the short arm of chromosome 11, which has an integral role in cell-to-cell adhesion as well as extracellular matrix interactions [22]. It is expressed in a standard form (CD44s) and as a numerous splice variant (CD44v) [23]. CD44 expression has been identified in a variety of tissues and malignant tumors [24], while CD44 variant isoforms are thought to interfere with cellular growth and metastatic potential of various tumors [21]. It is thought that invasive and metastatic potential can be mediated through cell surface CD44 with hyaluronan [11]. Moreover, this is not the first time CD44 and clinical prognosis of cancer patients is observed [6]. High levels of this marker were reported in gastric, colorectal, and non-small-cell lung cancer [25, 26]. In our study, as expected, expression of CD44 was not correlated to patient's demographics. However, there was very strong evidence of association between CD44 and lymph nodal metastasis (*p* = 0.00). Several studies have ended with equivocal results, presumably due to slight differences in specificity between various antibodies or the use of different techniques. It is also argued that the existence of numerous CD44 isoforms leads to increased cross-reactivity between the antibodies, resulting in conflicting data [24, 27]. On our study, on univariate analysis, CD44 staining had a significant correlation with positive lymph nodal disease, high tumor grade, and perineural/microvascular invasion.

Ki67 is a nuclear proliferation-associated antigen. Thus, it is expressed in both the growth and synthesis phases of the cell cycle but not in the resting phase [5]. As a result of the above, this antigen can be used to estimate the proportion of active cells over the cell cycle. Various epitopes of the Ki67 antigen (Ki67, KiSI, Ki55, and MIB-1) have been utilized to measure proliferation of different tumors. It is well-known that Ki67 expression in PDAC correlates with tumor grade and positive nodal status. Moreover, previous studies support that expression of Ki67 is potentially prognostically significant if exceeding 5% [28]. Our results show that Ki67 is related to higher tumor grade, positive lymph nodal status, and ultimately higher disease stage (*p* ≤ 0.01).

New molecular markers are under investigation aiming at implementing targeted therapies in the future. The authors are planning to proceed with a new prospective trial on PDAC patients, regarding the expression of MAP4K5, which started being studied very recently [29].

Conclusively, vimentin+/CD44+/high Ki67 expressions were correlated with poor clinical outcome. As a future direction, targeting these markers with novel therapeutic agents could potentially have valuable role in the so far poor PDAC treatment armamentarium.

## Conflicts of Interest

The authors declare that there is no conflict of interest regarding the publication of this paper.

## Figures and Tables

**Table 1:** Primary antibodies: characteristics and applied dilution.

Antigen	Antibody	Clone	Dilution	Commercial product used
Vimentin	Monoclonal mouse Anti-vimentin	V9	1 : 100	DAKO
Ki67	Monoclonal mouse Anti-human Ki67 antigens	M1B1	1 : 100	DAKO
CD44	Monoclonal mouse Anti-human	DF1485	1 : 100	DAKO

**Table 2:** Demographic and histopathological characteristics of included patients.

Characteristics	
Sex (female)^∗^	15 (28.3%)
Age (years)^†^	68 (44–82)
Operation	
Classic Whipple's^∗^	12 (22.6%)
PPPD^∗^	31 (58.5%)
Distal pancreatectomy^∗^	10 (18.9%)
T stage	
T1^∗^	4 (7.5%)
T2^∗^	9 (17%)
T3^∗^	31 (58.5%)
T4^∗^	9 (17%)
N stage	
N0^∗^	11 (20.8%)
N1^∗^	42 (79.2%)
Size of primary tumor (cm)^†^	4.1 (1.2–7.1)
Histopathological grade	
I^∗^	8 (15.1%)
II^∗^	17 (32.1%)
III^∗^	24 (45.3%)
IV^∗^	4 (7.5%)
Lymphovascular invasion^∗^	37 (69.8%)
Peri/intraneural invasion^∗^	35 (66%)
Resection margin	
R0^∗^	41 (77.4%)
R1^∗^	10 (18.9%)
R2^∗^	2 (3.8%)

^∗^Number (%); ^†^median (minimum–maximum); PPPD: pylorus preserving pancreaticoduodenectomy.

**Table 3:** Expression rates of the studied markers.

Marker	*n* (%)
CD44	
Low	12 (22.6%)
Moderate	8 (15.1%)
High	33 (62.3%)
Ki67	
Nil	1 (1.9%)
Low	23 (43.4%)
High	29 (54.7%)
Vimentin	
Negative	21 (39.6%)
Positive	32 (60.4%)

**Table 4:** Correlation of marker expression with survival.

Protein	*p* value^∗^
CD44	0.305
Ki67	0.174
Vimentin	0.029

^∗^Log-rank (Mantel-Cox) test for correlation with overall survival.

**Table 5:** Correlations between studied biomarkers.

CD44 with Ki67	*p* = 0.022
Vimentin with Ki67	*p* < 0.001
CD44 with Ki67	*p* = 0.015
